# Applications of Artificial Intelligence in Alpha-1 Antitrypsin Deficiency: A Systematic Review from a Respiratory Medicine Perspective

**DOI:** 10.3390/medicina61101768

**Published:** 2025-09-30

**Authors:** Manuel Casal-Guisande, Laura Villar-Aguilar, Alberto Fernández-Villar, Esmeralda García-Rodríguez, Ana Casal, María Torres-Durán

**Affiliations:** 1 Fundación Pública Galega de Investigación Biomédica Galicia Sur, Hospital Álvaro Cunqueiro, 36312 Vigo, Spain; 2 NeumoVigo I+i Research Group, Galicia Sur Health Research Institute (IIS Galicia Sur), SERGAS-UVIGO, 36312 Vigo, Spain; laura.villar.aguilar@sergas.es (L.V.-A.); alberto.fernandez.villar@sergas.es (A.F.-V.); maria.esmeralda.garcia.rodriguez@sergas.es (E.G.-R.); ana.casal.mourino@sergas.es (A.C.); maria.luisa.torres.duran@sergas.es (M.T.-D.); 3 Centro de Investigación Biomédica en Red, CIBERES ISCIII, 28029 Madrid, Spain; 4 Pulmonary Department, Hospital Álvaro Cunqueiro, 36312 Vigo, Spain; 5 School of Industrial Engineering, University of Vigo, 36310 Vigo, Spain

**Keywords:** alpha-1 antitrypsin deficiency, chronic obstructive pulmonary disease, emphysema, liver, hepatology, artificial intelligence, machine learning

## Abstract

*Background and Objectives*: Alpha-1 antitrypsin deficiency (AATD) is a rare genetic condition associated with chronic respiratory diseases such as chronic obstructive pulmonary disease (COPD) and emphysema, and with liver involvement through a distinct toxic gain-of-function mechanism. Despite its clinical relevance, AATD remains underdiagnosed and exhibits marked phenotypic heterogeneity. Artificial intelligence (AI) has shown growing potential in respiratory medicine, yet its application to AATD is still limited. This systematic review synthesizes the clinical evidence on AI in AATD, primarily in the respiratory domain and, where available, in hepatic outcomes. *Materials and Methods*: We conducted a PRISMA-guided search (PubMed, Web of Science, IEEE Xplore) for original, peer-reviewed articles (January 2014–September 2025) applying AI to detection, classification, stratification, or prediction tasks in AATD. *Results*: Six studies met eligibility criteria. Supervised models (e.g., XGBoost, penalized regression, Transformer-based architectures) and one unsupervised approach were identified. Applications included screening in COPD populations, prediction of emphysema progression from CT, proteomic modeling of lung function, identification of clinical subgroups, and prediction of clinical outcomes in AATD-associated liver disease. External validation and genotype diversity remained limited across studies. *Conclusions*: Although AI shows promise in improving detection, prognosis, and patient stratification in AATD across both respiratory and hepatic manifestations, the current evidence remains limited. Broader, multicenter validation in genotype-diverse cohorts is required to confirm its clinical utility and support the implementation of precision medicine in AATD.

## 1. Introduction

Alpha-1 antitrypsin deficiency (AATD) is a rare genetic condition inherited in a codominant manner, caused by mutations in the SERPINA1 gene. It is characterized by reduced circulating levels of alpha-1 antitrypsin (AAT), a glycoprotein with essential functions in protecting lung tissue [1,2]. AAT deficiency increases the risk of chronic obstructive pulmonary disease (COPD), emphysema, and other respiratory diseases, particularly among individuals exposed to tobacco smoke. Beyond the lungs, AATD may also affect the liver in both adults and children [3,4] and, more rarely, the skin (necrotizing panniculitis) or present as a vasculitis.

The underlying mechanisms differ by organ. In the respiratory system, disease results from a toxic loss of function. This occurs because AAT levels are insufficient to inhibit neutrophil elastase and other proteases. In contrast, liver involvement reflects a toxic gain of function. It is characterized by intracellular accumulation and polymerization of misfolded AAT within the hepatocyte endoplasmic reticulum, particularly in genotypes such as Pi*ZZ. This process ultimately leads to cellular stress, inflammation, and progressive liver injury [1,2,3,4].

From a clinical standpoint, the management of AATD represents a significant challenge due to its high rate of underdiagnosis, considerable heterogeneity, and marked interindividual variability in disease progression [5]. Despite diagnostic advances and the availability of specific treatments—such as augmentation therapy—many patients remain undiagnosed or receive the diagnosis only at advanced stages. By then, irreversible loss of pulmonary function is often already present. Although some studies have identified factors associated with functional decline or exacerbation frequency, the available evidence remains fragmented and inconsistent, reflecting the clinical complexity of AATD [6,7,8].

In this context, artificial intelligence (AI) has emerged as a powerful tool with high potential to address diagnostic and prognostic challenges in chronic respiratory diseases. Machine learning-based techniques have demonstrated utility in various clinical tasks. These include patient classification, early disease detection, prediction of clinical events, and identification of relevant clinical subgroups in conditions such as COPD, asthma, and sleep apnea [9,10,11,12]. However, its specific application in the context of AATD remains limited and poorly systematized.

Among the most prominent initiatives aimed at improving knowledge of AATD is the European Alpha-1 Research Collaboration (EARCO) project, launched in 2020 with the goal of promoting clinical and educational research in this field. One of the pillars of this consortium is the development of an international registry that includes patients carrying genetic variants associated with AATD—including Z, S, null, and rare alleles, both in homozygous and compound heterozygous states. This registry systematically collects longitudinal data through structured periodic visits [13]. The existence of this standardized multicenter database provides an ideal framework for the implementation of AI models. It enables large-scale analysis and fosters a more precise and personalized approach to this genetic condition within respiratory medicine.

Despite this potential, to date, no systematic review has specifically explored the use of AI in the clinical study of AATD, with an emphasis on its respiratory manifestations. The aim of this review is therefore to identify, analyze, and synthesize the published evidence over the past decade regarding the application of AI techniques in the clinical context of AATD. Special attention is given to the methodological approaches used, types of data employed, algorithms applied, and their practical relevance in tasks such as disease detection, classification, and prediction.

## 2. Why Artificial Intelligence?

Since its inception, AI has been defined as a field aimed at developing systems capable of behaving in a rational and “intelligent” manner, emulating human abilities such as learning, problem-solving, perception, and decision-making. In the biomedical domain, AI has experienced remarkable expansion over the past decade, driven by the massive digitization of clinical data, improvements in computational capacity, and the advancement of increasingly sophisticated algorithms. These developments have enabled its application to tasks such as automated diagnosis, prediction of adverse events, risk stratification, and the design of personalized medicine strategies.

AI encompasses a wide range of approaches, from expert systems based on rules—which are capable of reproducing predefined clinical reasoning—to models that learn automatically from data. While the former relies on structured knowledge, the latter can identify complex patterns in large volumes of heterogeneous data.

In the field of respiratory diseases, AI has already proven useful in various clinical applications, such as population screening, early disease detection, clinical cluster identification, exacerbation prediction, and automated interpretation of radiological images, particularly in conditions like COPD, asthma, and sleep apnea [9,10,11,12]. In the case of AATD, its use remains incipient but holds promising prospects. The considerable heterogeneity of the disease, diagnostic underrecognition, low prevalence, and the need to integrate clinical, genetic, functional, and imaging data make AATD a particularly suitable scenario for benefiting from AI-based approaches.

Although the field of AI includes multiple branches, this review focuses specifically on Machine Learning approaches, as these are the most employed in the current clinical literature on AATD. For the sake of clarity and readability, we provide a detailed overview of ML strategies in Appendix A.

## 3. Methods

A systematic review of the literature published between January 2014 and September 2025 was conducted, following the PRISMA (Preferred Reporting Items for Systematic Reviews and Meta-Analyses) guidelines [14]. The time frame was set from 2014 onwards, as this period marks the point at which AI methods began to be more consistently translated into clinical research and practice, with a rapid increase in healthcare-related applications and publications. The objective was to identify studies that applied AI techniques to the clinical management of AATD, with a particular focus on its respiratory manifestations.

The literature search was performed in three databases: PubMed, Web of Science, and IEEE Xplore. In PubMed, we used only free-text terms restricted to the Title/Abstract fields to improve precision in a field with interdisciplinary technical terminology. In Web of Science, we used the topic field and in IEEE Xplore we searched all metadata. The full, reproducible strategies are provided in Appendix B (Appendix B.1, Appendix B.2 and Appendix B.3).

Only original, peer-reviewed publications written in English were included. Reviews, letters to the editor, brief communications, editorials, and conference proceedings or preprints were excluded. Studies focusing exclusively on animal models or without direct relevance to the clinical context of AATD were also excluded.

Eligible studies had to apply artificial intelligence methods to clinical tasks such as detection, classification, stratification, or prediction in patients with AATD, regardless of the type of data used (clinical, functional, genetic, imaging, or demographic). Studies that relied solely on conventional statistical methods, such as simple logistic regression, without incorporating ML or AI methodologies, were excluded. Studies applying AI techniques to contexts unrelated to the clinical care of AATD (e.g., general hepatology without genetic AATD, cardiovascular or renal conditions) were excluded.

Study selection was performed independently by two reviewers (M.C.-G. and L.V.-A.) in two successive phases: initial screening by title and abstract, followed by full-text review. Discrepancies were resolved by consensus or through the intervention of a third reviewer (M.T.-D.).

For each included study, data were extracted on general design characteristics, population analyzed, algorithms used, predictive variables, and sample size.

Due to the heterogeneity observed among studies in terms of design, data sources, techniques employed, and evaluation metrics, conducting a meta-analysis was not deemed feasible. Likewise, the application of a structured quality assessment tool (e.g., PROBAST or related instruments) was not considered appropriate, as the exploratory scope and methodological variability of the included studies prevented consistent scoring across domains. Instead, a structured narrative synthesis was undertaken, organized according to methodological approaches and clinical applications of the AI models identified.

## 4. Results

### 4.1. Study Selection

**Figure 1:**
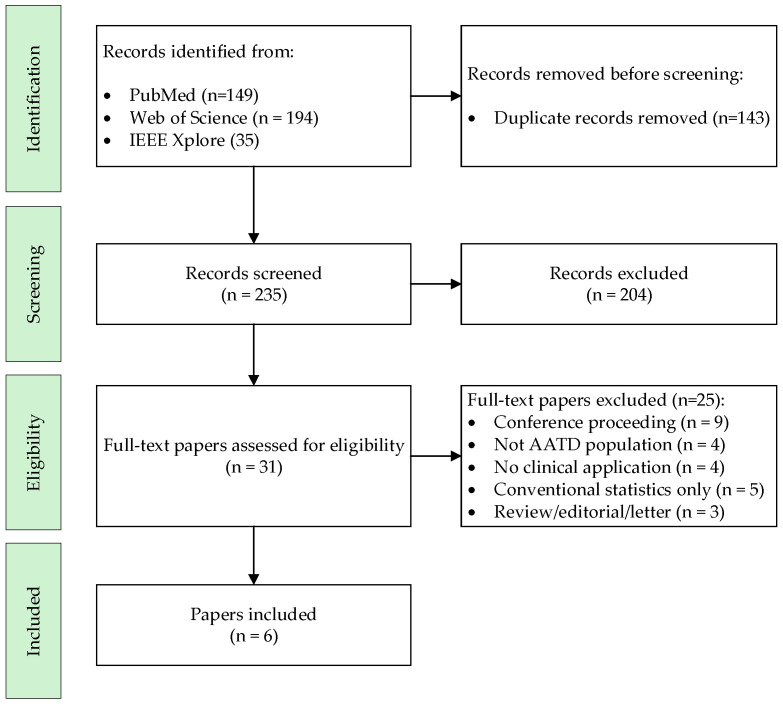
Flow Diagram of the Systematic Review.

The systematic search strategy identified a total of 235 studies. After applying the inclusion and exclusion criteria, six studies were ultimately selected for inclusion in the review, as detailed in Figure 1 (PRISMA diagram). Most of the excluded full-text articles fell into the following categories: conference proceedings (n = 9), studies not conducted in AATD populations (n = 4), works without a clinical application of AI (n = 4), studies relying solely on conventional statistical methods (n = 5), and reviews or editorials (n = 3).

Table 1 summarizes the main characteristics of the included studies.

### 4.2. General Characteristics of the Included Studies

The six selected studies were published between 2022 and 2025. Most utilized data from large cohorts in the United States and the United Kingdom (such as COPDGene, AATD GMS, UK Biobank, or AATD UK Registry) [15,16,17,18,20]. In addition, one study was based on a hospital cohort of AATD patients followed at a specialized monographic clinic in Spain (Hospital Álvaro Cunqueiro, Vigo) [19]. While five studies focused on respiratory applications, one addressed clinical outcomes in AATD-associated liver disease using UK Biobank data [20].

Sample sizes ranged from ~200 to >21,000 individuals, with one study analyzing 11,583 liver-disease patients including 455 with AATD liver disease [20]. Variables spanned plasma biomarkers, standard clinical features (age, sex, smoking, lung function, augmentation therapy), thoracic CT imaging, and liver-related laboratory indices, as well as electronic health record (EHR) and self-reported data.

Regarding AI approaches, five studies applied supervised algorithms (e.g., XGBoost, penalized regression/elastic net, random forest, Transformer-based architectures), and one used unsupervised learning (k-prototypes clustering). All studies included explicit validation procedures (cross-validation and/or independent test sets).

### 4.3. Clinical Applications of Artificial Intelligence in Respiratory AATD

#### 4.3.1. Screening and Early Detection of AATD in Respiratory Populations

The study by Pfeffer et al. [17] addressed the issue of AATD underdiagnosis through the development of a predictive model applied to a large real-world database (EVERSANA) with over 21,000 COPD patients. Using the XGBoost algorithm and more than 500 clinical and laboratory variables, the model improved the identification of individuals with a high probability of having AATD, achieving an AUROC of approximately 0.90 with robust cross-validation. Feature importance analysis using SHAP confirmed the contribution of diagnostic codes, medication history, and laboratory values.

#### 4.3.2. Prediction of Structural Emphysema Progression

Curiale and San José Estépar [16] developed LobTe, a Transformer-based architecture adapted for medical imaging and longitudinal data. Transformers employ attention mechanisms that weigh the relative importance of different input features, allowing the model to focus on the most informative regions. In LobTe, each CT scan was partitioned by pulmonary lobe, providing a spatial encoding that preserved anatomical structure. The model also integrated temporal encoding of repeated scans, enabling it to capture how emphysema progressed across time. This combination—referred to as spatio-temporal encoding—allowed the network to jointly model the distribution of emphysema across lobes and its longitudinal evolution, improving prediction of annual change in lung density. In validation, the LobTe model achieved a mean absolute error of 0.57 g·L^−1^ and a Pearson correlation of ρ = 0.61 between predicted and observed progression, outperforming baseline CNN, LSTM, and TabNet comparators.

#### 4.3.3. Biomarker-Based Modeling of Lung Function

Two studies applied AI-based approaches using biomarkers to estimate pulmonary function in AATD patients:

- Moll et al. [15] developed a proteomic score (protRS) predictive of the FEV_1_/FVC ratio, validated in both healthy individuals (Pi*MM) and those with AATD (Pi*ZZ). The model achieved cross-validated AUROCs above 0.80 for distinguishing airflow obstruction and highlighted proteins with potential diagnostic and therapeutic value, such as AGER.
- Spittle et al. [18] applied conventional multivariable regression models to explore associations between plasma proteins and lung function (FEV_1_ and TLCO) in 111 Pi*ZZ patients with COPD from the AATD UK Registry. Their models identified CC16 as a predictor of TLCO decline, with explanatory power improving from R^2^ = 0.02 (clinical variables only) to R^2^ = 0.13 when biomarkers were added.

#### 4.3.4. Clustering Approaches

Villar-Aguilar et al. [19] employed unsupervised learning techniques (k-prototypes algorithm) to identify five clinically distinct subgroups within a hospital cohort of AATD patients. The optimal cluster solution was determined using the elbow method, and the resulting groups differed in symptoms, functional patterns, and radiological phenotype, offering a clinically interpretable basis for more personalized stratification.

#### 4.3.5. Hepatic Outcomes in AATD

Meng et al. [20] developed a stacking ensemble (combining tree-based learners, regularized regression and neural networks) to predict all-cause mortality, liver-related death and liver transplant among patients with AATD-associated liver disease in UK Biobank. Using 58 predictors (demographics, comorbidities, lifestyle, and laboratory/spirometry indices), the model achieved AUROCs ranging from 0.68 to 0.91 depending on the outcome, highlighting the potential of AI to inform risk stratification and surveillance in the hepatic compartment of AATD.

## 5. Discussion

### 5.1. Main Findings

This systematic review identified six original studies applying AI to AATD across respiratory and hepatic domains. Reported applications encompassed screening within COPD populations, prediction of emphysema progression from CT, proteomic modeling of lung function, unsupervised patient stratification, and risk prediction in AATD-associated liver disease. Most studies employed supervised approaches—such as gradient boosting, penalized regression, or Transformer-based architectures. Only one study used unsupervised learning to identify clinically distinct subgroups. One included study (Spittle et al. [18]) used conventional multivariable regression. Although not strictly an AI method, its inclusion reflects how “predictive modeling” is sometimes framed in the AATD literature. It also illustrates that, in this rare-disease context, classical statistical approaches remain common.

Performance metrics aligned with task type. For classification, AUROC values ranged from ~0.68 to 0.91 depending on outcome (e.g., case-finding and hepatic endpoints). For regression, models reported mean absolute error and/or correlation (e.g., ρ ≈ 0.61 for emphysema-progression prediction). In one conventional regression analysis, R^2^ was reported for TLCO decline. Unsupervised clustering was evaluated by internal criteria and clinical interpretability rather than predictive metrics.

### 5.2. Methodological Strengths

Across studies, internal validation procedures were generally appropriate, variable selection was explicit, and several analyses leveraged large, well-characterized cohorts. Multimodal integration—combining clinical, spirometric, imaging, and proteomic data—was used to capture complementary signals relevant to AATD. Methodological innovation was evident in the use of a lobe-based spatio-temporal Transformer for longitudinal CT (Curiale and San José Estépar [16]). Another example was a stacking ensemble that combined tree-based, linear, and neural models for hepatic outcomes (Meng et al. [20]). Collectively, these choices illustrate mature ML practices and the potential for improved predictive performance compared with conventional approaches.

### 5.3. Limitations of the Included Studies

Important limitations persist at the primary-study level. Most models lacked external validation in independent cohorts, restricting applicability in real-world settings. Interpretability was often limited, with few analyses clearly identifying the most influential variables driving predictions. The predominance of retrospective designs and the scarce incorporation of longitudinal data also constrain the ability to model trajectories or treatment responses. Heterogeneity in objectives, data sources, and variable definitions also hinders direct comparison across models.

Genotypic diversity was limited. Although some studies included less severe variants (e.g., Pi*SZ), their representation was small, and subgroup-specific analyses were uncommon. This reduces robustness across the full spectrum of AATD. Finally, while one study examined hepatic outcomes [20], rare events (e.g., transplant) and the small number of confirmed Pi*ZZ cases further constrain the generalizability of those findings.

### 5.4. Clinical Implications

In line with respiratory-society guidance, adults with persistent airflow obstruction—particularly those with COPD—should be tested for AATD [1,21]. However, its under-recognition remains substantial. Classic population-screening data from St. Louis suggested a large gap between expected and identified Pi*ZZ individuals [22,23], motivating scalable approaches to prioritize testing. In this context, AI-enabled case-finding in broad respiratory populations (e.g., Pfeffer et al. [17]) can help operationalize guideline-based screening.

The studies included indicate that AI can:

- improve screening of AATD within general respiratory populations;
- predict structural emphysema progression or lung-function change to support personalized monitoring;
- identify clinical subgroups with distinct profiles that may inform tailored strategies;
- support risk stratification and longitudinal surveillance in AATD-associated liver disease by integrating demographics, lifestyle, and laboratory indices. This may complement guideline-based monitoring and facilitate co-management between hepatology and pulmonology, particularly for Pi*ZZ and compound heterozygotes.

These applications align with personalized medicine and may enhance both early diagnosis and clinical decision-making in AATD.

#### 5.4.1. Practical Implementation and Workflow Integration

Several of the AI applications identified in this review can already be envisioned within everyday clinical routines:

- EHR-triggered case-finding: triage models running on routinely collected COPD data to prioritize AAT measurement and/or genotyping, with explicit threshold selection (balancing sensitivity, specificity, and downstream capacity), periodic calibration, and concise feature-attribution summaries for clinical review.
- CT-based monitoring of emphysema progression: standardized pipelines—quality-assured acquisition, automated lobar parsing, and density extraction—feeding spatio-temporal models that estimate annual change in lung density and flag fast progressors; site protocols should define imaging intervals, radiation considerations, and quality assurance
- Biomarker-assisted follow-up: curated protein panels to anticipate lung-function decline, with pre-analytical standards and reporting aligned to clinical laboratory practice.
- Hepatic risk stratification: risk scores integrating demographics, lifestyle, and liver-related indices to prioritize surveillance intensity and enable shared hepatology–pulmonology care in higher-risk genotypes.

Across scenarios, external validation, model versioning, monitoring for data drift, recalibration, and transparent outputs are essential to support clinician trust and safe adoption.

#### 5.4.2. Adoption Barriers and Enablers

Barriers include small sample sizes, limited genotype diversity, heterogeneous data sources, and scarce prospective evaluation. Enablers include access to multicenter registries (e.g., EARCO), harmonized data dictionaries and imaging protocols, and clinician-in-the-loop deployments with pre-specified performance targets and calibration plans. Given the rarity of AATD, shared infrastructures and privacy-preserving learning (e.g., federated or semi-supervised strategies) may facilitate scale-up while safeguarding patient confidentiality.

### 5.5. Future Opportunities and Need for Further Analysis

Compared with other chronic respiratory diseases, the penetration of AI in AATD remains limited. This creates an opportunity to develop tools targeted to this condition. Collaborative infrastructures such as EARCO, which integrate clinical, genetic, functional, and imaging data across centers, are well positioned to:

- train and externally validate predictive models on high-quality, diverse datasets;
- conduct longitudinal analyses of clinical and structural progression;
- compare trajectories across genotypes (Pi*ZZ, Pi*SZ, Pi*MZ);
- evaluate individualized responses to augmentation therapy or other treatments.

Leveraging these datasets can accelerate progress toward precision medicine and enable the standardization of validated predictive models in multicenter contexts.

### 5.6. Limitations of This Review

This review has limitations. The small number of eligible studies and their methodological heterogeneity precluded meta-analysis. Although strict inclusion criteria were applied, some relevant studies may have been missed due to insufficient methodological detail in titles and abstracts. Conference proceedings and preprints were excluded a priori; exploratory searches indicated that available abstracts mentioning AATD and AI were scarce and of marginal clinical relevance. The search was restricted to English-language publications, which may introduce language bias.

Finally, given the exploratory scope and heterogeneity of included studies, a structured quality-assessment tool (e.g., PROBAST) was not applied. No formal inter-rater statistics were computed for screening and data extraction; discrepancies were infrequent and resolved by consensus, but this remains a methodological limitation.

Despite broadened searches across PubMed, Web of Science, and IEEE Xplore, the number of eligible studies remained very low. This underscores both the paucity of published AI applications in clinical AATD and the need for larger, genotype-diverse, prospectively validated work.

## 6. Conclusions

The evidence synthesized in this systematic review shows that artificial intelligence holds promising potential in the clinical management of AATD across respiratory and hepatic manifestations. However, the number of published studies remains very low, underscoring that the field is still emerging. Existing applications range from screening to functional prediction, clustering of clinical subgroups, and risk stratification of liver disease. Future studies should prioritize external validation in genotype-diverse, multicenter, and multiethnic cohorts, with harmonized protocols and local calibration, to secure generalizability and equitable clinical performance.

## Figures and Tables

**Table 1:** Study results.

Study	Objective	Size	Variables Used	Models and Methodological Approach
Moll et al. [15]	Investigate associations between plasma protein biomarkers and lung function and build a protein risk score to predict COPD in individuals with AATD (Pi*ZZ)	N = 4798; participants from COPDGene (Pi*MM = 4446) and AAT Genetic Modifiers Study (Pi*ZZ = 352)	Proteomic panels (SomaScan/Olink), spirometry (FEV1/FVC), demographic variables (age, sex, smoking) and augmentation therapy	Multivariable regression and linear mixed models to identify biomarker–function associations; development/validation of a protein risk score (protRS); Random Forest for ranking protein importance; internal cross-validation with AUROC comparisons
Curiale and San José Estépar [16]	Develop a model to predict annual emphysema progression in AATD patients using longitudinal chest CT data	N = 4821 COPDGene participants; training set: 2802 Pi*MM; test set: 2019 (Pi*MM = 1983, Pi*ZZ = 15, Pi*SZ = 18, Pi*MZ = 3)	High-resolution CT (per-lobe PERC15 density and lung volume), age, sex, smoking status, interval between scans and clinical metadata	LobTe, a lobe-based Transformer with spatial-temporal encoding; performance evaluated by mean absolute error and Pearson correlation (ρ = 0.61); comparisons with CNN, LSTM and TabNet; stratified cross-validation and independent test set
Pfeffer et al. [17]	Develop a real-world predictive model to identify AATD in COPD populations	N = 21,381 (AATD-positive = 13,585; AATD-negative = 7796) from the EVERSANA claims/EHR database (~10.4 M COPD records)	Administrative data: ICD-9/10 codes; prescribed medications; lab results (excluding AAT level); demographics; comorbidities and respiratory symptoms	XGBoost model (>500 features); cross-validation and hyperparameter tuning; feature importance via SHAP; reported AUROC ≈ 0.90
Spittle et al. [18]	Assess utility of plasma protein biomarkers for predicting lung function (FEV_1_, TLCO) decline in AATD patients	N = 200 patients from the AATD UK Registry; 167 had the Pi*ZZ genotype and 124 had COPD. Multivariable models were built on the 111 Pi*ZZ patients with COPD	Seven serum biomarkers (CRP, CCL18, CC16, IL-6, IL-8, TNF-α, SP-D) plus age, sex, smoking status, augmentation therapy and lung function	Multivariable regression; exploratory models with and without biomarkers; CC16 identified as predictor of TLCO decline
Villar-Aguilar et al. [19]	Identify clinically relevant subgroups of AATD patients using unsupervised learning	N = 210 AATD patients from a single-center cohort (Hospital Álvaro Cunqueiro, Vigo; EARCO registry)	Baseline demographic, clinical, functional and radiological variables collected under the EARCO protocol	k-prototypes algorithm for mixed data; optimal number of clusters determined by the elbow method; five clusters distinguished by emphysema severity, bronchiectasis, age/comorbidity profile and hepatic features
Meng et al. [20]	Develop a stacking-ensemble ML model to predict clinical outcomes in AATD-associated liver disease	N = 11,583 UK Biobank participants with liver disease; 455 had AATD-associated liver disease (including 20 Pi*ZZ genotypes)	Fifty-eight predictors: demographics, baseline comorbidities, lifestyle factors (alcohol intake, smoking), laboratory tests and spirometry	Stacking ensemble (Random Forest, XGBoost, LightGBM, Elastic Net, multilayer perceptron) with nested 5-fold cross-validation; feature selection preceding modeling; AUROC varied by outcome: mortality 0.68, liver-related death 0.76, liver transplant 0.91

Abbreviations: AATD, alpha-1 antitrypsin deficiency; PERC15, 15th percentile lung density on CT; protRS, protein risk score; TLCO, transfer factor for carbon monoxide; EHR, electronic health records; SHAP, Shapley Additive Explanations; EARCO, European Alpha-1 Research Collaboration; AUROC, area under the receiver-operating characteristic curve.

## Data Availability

The original contributions presented in this study are included in the article.

## Appendix A

Machine Learning represents one of the most dynamic and widely applied branches of current AI. Through algorithms capable of identifying patterns in data via iterative training, these models have transformed the way complex clinical problems are addressed. Depending on the type of data and the analytical objective, algorithms can be grouped into several categories [24]: supervised learning, semi-supervised learning, and unsupervised learning; reinforcement learning exists as a separate paradigm but has not been reported in AATD.

Supervised learning uses labeled datasets, where each patient record (e.g., clinical, spirometric, imaging or proteomic data) is associated with a known outcome. It can be applied to classification tasks, such as distinguishing individuals at high risk of AATD within large COPD cohorts, or to regression tasks, such as predicting continuous outcomes including longitudinal changes in lung density from CT scans or lung-function trajectories (e.g., FEV_1_ or TLCO) from biomarker and clinical data.

Semi-supervised learning combines a small labeled set with a larger unlabeled pool, propagating information from confirmed labels to unlabeled examples. This is particularly relevant in rare diseases such as AATD: only a limited number of patients have confirmed genotypes (e.g., Pi*ZZ), whereas far larger numbers of COPD cases remain ungenotyped. Semi-supervised methods could exploit both groups simultaneously, thereby improving case-finding, risk stratification, and model generalizability. Although no included study has yet used this approach, it represents a promising direction for future research in AATD.

Unsupervised learning does not require labels; instead, algorithms search for hidden structures within the data. In AATD, this can help to identify natural subgroups of patients with distinct emphysema severity, comorbidity patterns, or hepatic involvement, and to simplify complex datasets through dimensionality reduction.

Beyond these paradigms, ensemble methods constitute a transversal strategy to increase robustness and accuracy by combining multiple learners. They can be applied to enhance screening, support biomarker-based prediction, or stratify clinical outcomes in AATD.

The versatility of these approaches, together with their ability to integrate multimodal data and handle large volumes of information, positions ML as a particularly promising avenue for optimizing diagnosis, monitoring, and treatment in AATD.

## Appendix B

### Appendix B.1. PubMed Search Strategy

In PubMed, we used free-text terms restricted to the Title/Abstract fields to maximize precision in a context of heterogeneous interdisciplinary terminology. The complete query was:
(“alpha-1 antitrypsin deficiency”[Title/Abstract] OR “alpha-1 antitrypsin”[Title/Abstract] OR “SERPINA1”[Title/Abstract] OR “AATD”[Title/Abstract] OR “AAT”[Title/Abstract] OR “PiZZ”[Title/Abstract] OR “PiSZ”[Title/Abstract] OR “PiMZ”[Title/Abstract] OR “Z allele”[Title/Abstract] OR “EARCO”[Title/Abstract]) AND (“artificial intelligence”[Title/Abstract] OR “machine learning”[Title/Abstract] OR “deep learning”[Title/Abstract] OR “neural network”[Title/Abstract] OR “logistic regression”[Title/Abstract] OR “support vector machine”[Title/Abstract] OR “random forest”[Title/Abstract] OR “XGBoost”[Title/Abstract] OR “decision tree”[Title/Abstract] OR “k-means”[Title/Abstract] OR “hierarchical clustering”[Title/Abstract] OR “predictive model”[Title/Abstract] OR “ensemble learning”[Title/Abstract] OR “k-nearest neighbors”[Title/Abstract] OR “KNN”[Title/Abstract]) AND (“2014”[Date—Publication]: “2025”[Date—Publication])

### Appendix B.2. Web of Science Search Strategy

In Web of Science (WoS), the search was performed in the Topic (TS) field, which includes title, abstract, author keywords, and Keywords Plus. The same combination of AATD and AI/ML terms was applied. The complete query was:
TS = ((“alpha-1 antitrypsin deficiency” OR “alpha-1 antitrypsin” OR SERPINA1 OR AATD OR AAT OR PiZZ OR PiSZ OR PiMZ OR “Z allele” OR EARCO) AND (“artificial intelligence” OR “machine learning” OR “deep learning” OR “neural network” OR “logistic regression” OR “support vector machine” OR “random forest” OR XGBoost OR “decision tree” OR “k-means” OR “hierarchical clustering” OR “predictive model” OR “ensemble learning” OR “k-nearest neighbors” OR KNN))

The timespan was restricted to 2014–2025, and only records classified as Article were retained. Conference proceedings, meeting abstracts, and other non-article formats were excluded.

### Appendix B.3. IEEE Xplore Search Strategy

In IEEE Xplore, the search was conducted across All Metadata, again using the same AATD and AI/ML blocks. The complete query was:
(“alpha-1 antitrypsin deficiency” OR “alpha-1 antitrypsin” OR SERPINA1 OR AATD OR AAT OR PiZZ OR PiSZ OR PiMZ OR “Z allele” OR EARCO) AND (“artificial intelligence” OR “machine learning” OR “deep learning” OR “neural network” OR “logistic regression” OR “support vector machine” OR “random forest” OR XGBoost OR “decision tree” OR “k-means” OR “hierarchical clustering” OR “predictive model” OR “ensemble learning” OR “k-nearest neighbors” OR KNN)
